# Draft Genome Sequences of Three Closely Related Isolates of the Purple Nonsulfur Bacterium *Rhodovulum sulfidophilum*

**DOI:** 10.1128/genomeA.00029-17

**Published:** 2017-03-16

**Authors:** Michael S. Guzman, Beau McGinley, Natalia Santiago-Merced, Dinesh Gupta, Arpita Bose

**Affiliations:** Department of Biology, Washington University in St. Louis, St. Louis, Missouri, USA

## Abstract

We report here the draft genome sequences of three isolates of *Rhodovulum sulfidophilum* from a single population that will serve as a model system for understanding genomic traits that underlie metabolic variation within closely related marine purple nonsulfur bacteria in natural microbial communities.

## GENOME ANNOUNCEMENT

*Rhodovulum sulfidophilum* is a metabolically versatile, purple, nonsulfur bacterium commonly isolated from marine habitats and hypersaline environments (1, 2). *R. sulfidophilum* is used as a model organism to study the mechanisms underlying anoxygenic photosynthesis (3–5), oxidative sulfur metabolism (6), extracellular DNA and RNA production (7), and, recently, biohydrogen production (8). Despite this interest, there are few genomic resources for *R. sulfidophilum*, and, to date, only two strains have genome sequences (*R. sulfidophilum* DSM 2351 [9] and *R. sulfidophilum* DSM 1374 [10]). To gain a better understanding of the genomic diversity within *R. sulfidophilum* and to develop new marine models for studying anoxygenic phototrophic metabolism, we generated draft genome sequences of three *R. sulfidophilum* environmental isolates (Table 1) from a microbial mat in a brackish estuary in the Truck River near Woods Hole, Massachusetts, USA.

**TABLE 1:** Genome statistics and accession numbers for three isolates of *R. sulfidophilum*

Strain	No. of reads	Assembly size (Mb)	No. of contigs	GenBank accession no.
AB14	2,557,748	4.35	9	MSYP00000000
AB26	3,402,927	4.38	3	MSYQ00000000
AB30	1,835,746	4.25	16	MSYR00000000

Genomic DNA from each organism was isolated from a mid-log-phase culture grown in Difco marine broth 2216 (BD Diagnostic Systems, Sparks, MD, USA) using the DNeasy blood and tissue kit (Qiagen, Düsseldorf, Germany). Illumina 250-bp paired-end sequencing libraries were prepared using the Nextera sample prep kit (Illumina Inc., San Diego, CA, USA) and were sequenced on an Illumina MiSeq platform using V2 chemistry (Illumina, Inc., San Diego, CA, USA). Sequencing reads were quality- and adapter-trimmed using Trimmomatic version 0.33 (11) with the program’s default parameters for paired-end reads. The processed reads were *de novo* assembled using the CLC Genomics Workbench (CLC Bio-Qiagen, Aarhus, Denmark). Scaffolds were generated using the reference-based scaffolder MeDuSa (12) with *R. sulfidophilum* DSM 2351 as a guide for alignment. Reads were aligned to the DSM 2351 reference using the Bowtie2 version 2.2.29 (13) short-read mapper. Gene modeling and annotation was performed using the RAST version 2.0 (14–16) annotation pipeline. The whole-genome alignment was accomplished with LASTZ version 1.02.00 (17).

Each genome contains canonical genes involved in photolithoautotrophic metabolism, including photosynthetic genes in a photosynthetic gene cluster (18), form I and form II ribulose-1,5-bisphosphate carboxylase/oxygenase (RuBisCO) for carbon dioxide fixation, and genes involved in sulfur oxidation encoded by the Sox system (*soxXYZABCD*) completely conserved between strains. Whole-genome alignment revealed that the strains are closely related to DSM 2351 and DSM 1374, whereas only 57.8% (AB26), 78.7% (AB14), and 77.9% (AB30) of the sequence reads mapped to the *R. sulfidophilum* DSM 2351 genome. Scaffolding revealed contigs from AB14 aligned to plasmid 1, plasmid 2, and plasmid 3 of *R. sulfidophilum* DSM 2351, whereas AB30 only possessed sequences aligning to plasmid 1 and plasmid 2. Interestingly, strain AB26 contains sequences homologous to plasmid 3, in addition to a novel ~100-kb plasmid. This plasmid sequence contains a variety of genes for metal transport/metabolism, including nickel (*nikABCDE*), manganese (*sitABCD*), and zinc (*zuABC*) transport proteins. These results display new insights into the genomic diversity within closely related purple nonsulfur bacteria in marine ecosystems.

### Accession number(s).

The draft genome sequences have been deposited in GenBank under the accession numbers listed in Table 1.
